# Impact of variability in estimated glomerular filtration rate on major clinical outcomes: A nationwide population-based study

**DOI:** 10.1371/journal.pone.0244156

**Published:** 2020-12-17

**Authors:** Soojin Lee, Sehoon Park, Yaerim Kim, Yeonhee Lee, Min Woo Kang, Semin Cho, Yong Chul Kim, Seung Seok Han, Hajeong Lee, Jung Pyo Lee, Kwon Wook Joo, Chun Soo Lim, Yon Su Kim, Kyungdo Han, Dong Ki Kim

**Affiliations:** 1 Department of Internal Medicine, Seoul National University Hospital, Seoul, Korea; 2 Department of Internal Medicine, Seoul National University College of Medicine, Seoul, Korea; 3 Department of Biomedical Science. Seoul National University College of Medicine, Seoul, Korea; 4 Department of Internal Medicine, Armed Forces Capital Hospital, Gyeonggi-do, Korea; 5 Department of Internal Medicine, Keimyung University School of Medicine, Daegu, Korea; 6 Kidney Research Institute, Seoul National University College of Medicine, Seoul, Korea; 7 Department of Internal Medicine, Seoul National University Boramae Medical Center, Seoul, Korea; 8 Department of Statistics and Actuarial Science, Soongsil University, Seoul, Korea; Kaohsiung Medical University Hospital, TAIWAN

## Abstract

**Background:**

The estimated glomerular filtration rate (eGFR), commonly estimated using the serum creatinine value, often fluctuates throughout the serial measurement. The clinical significance of GFR variation among the general population with normal renal function has not yet been demonstrated. Thus, we explored the impact of GFR variability on adverse clinical outcomes.

**Methods:**

A nationwide retrospective cohort study using the Korean National Health Insurance System database was performed. National health screening examinees who underwent creatinine measurement ≥3 times between 2012 and 2016 were considered. Those with eGFR under 60 mL/min/m^2^ were excluded. The fluctuation of eGFR was represented with variability independent of the mean (VIM) index; which was calculated by the standard deviation divided by the exponent of the regression coefficient of the mean. Then, the risks of myocardial infarction (MI), stroke and death were assessed according to the quartiles of the VIM

**Results:**

Of total 3,538,500 participants, 0.29% of myocardial infarction (MI), 0.14% of stroke, 0.36% of deaths were observed during the median follow up of 3.27 years. Participants with the highest VIM index, which represents the highest eGFR variability, were significantly associated with an increased risk of MI (hazard ratio [HR]; 1.10, 95% confidence interval [95% CI]; 1.04–1.16), stroke (HR: 1.16; 95% CI 1.09–1.23), and death (HR: 1.18; 95% CI 1.12–1.24). The elevated risk of adverse events was consistent after the multivariate adjustment with potential confounding factors, except the risk of MI (HR 1.06; 95% 1.00–1.06).

**Conclusions:**

Increased eGFR variability exhibited an association with major clinical outcomes, indicating that monitoring eGFR variability might be a useful parameter for predicting the adverse outcomes.

## Introduction

The creatinine-based estimated glomerular filtration rate (eGFR) is the most widely used parameter to represent kidney function and stage the grade of chronic kidney disease (CKD) [1]. A single measured low eGFR exhibited an independent association with an increased risk of cardiovascular events, cardiovascular mortality, and all-cause mortality in CKD patients [2–4]. In the general population, a subtle decrease in eGFR within the normal range was also associated with cardiovascular and all-cause mortality [5]. However, in subjects with preserved kidney function, eGFR differs considerably even with a slight change in serum creatinine and may vary within the tests. Thus, further investigations are needed to interpret the significance of eGFR fluctuations.

Previous reports tried to investigate more suitable indicators to reflect the changes in eGFR, such as the rate of eGFR decline or eGFR trajectory, to assess the prognosis among the CKD patients [6–9]. However, the evidence is still uncertain about using the same indicators to predict outcomes among the general population whose renal function is relatively preserved. In this context, the fluctuation of several laboratory parameters was assessed and successfully predicted the risk of cardiovascular events and mortality in the healthy population [10–14].

Currently, more accessible medical services and regular national health check-ups enable patients and even healthy individuals to undergo periodic laboratory examinations, including kidney function tests. Serial eGFR measurements may facilitate clinicians to detect the decline in kidney function early; however, it is indeterminate for interpreting eGFR variations in the general population. The present study aimed to determine the clinical significance of the variability in multiple measurements of eGFR among individuals whose renal function is relatively preserved. We tried to examine whether eGFR variability could appropriately predict the risks of adverse outcomes and be applied to discriminate high-risk populations who require earlier intervention. Therefore, the current study was conducted using a nationwide, population-based dataset.

## Material and methods

### Ethical consideration

The study was approved by the Institutional Review Board of Seoul National University Hospital (IRB No. E-1801-027-913), and the designated government approved the approach to the database of the National Health Insurance System (NHIS). All authors followed the latest version of the Declaration of Helsinki throughout the study. As it was a retrospective study using fully anonymous and unidentifiable data without any additional intervention, informed consent was waived.

### Study population

A population-based cohort study was performed using the National Health Screening examination results obtained from the NHIS database [15]. The NHIS is a single organization managed by the Korean government and provides insured medical service to all Korean residents. The NHIS also offers the National Health Screening examination program, which includes general health check-ups and surveys, including lifestyle questionnaires. Non-office workers receive an annual examination, and the remaining office workers undergo a biannual examination.

**Fig 1:**
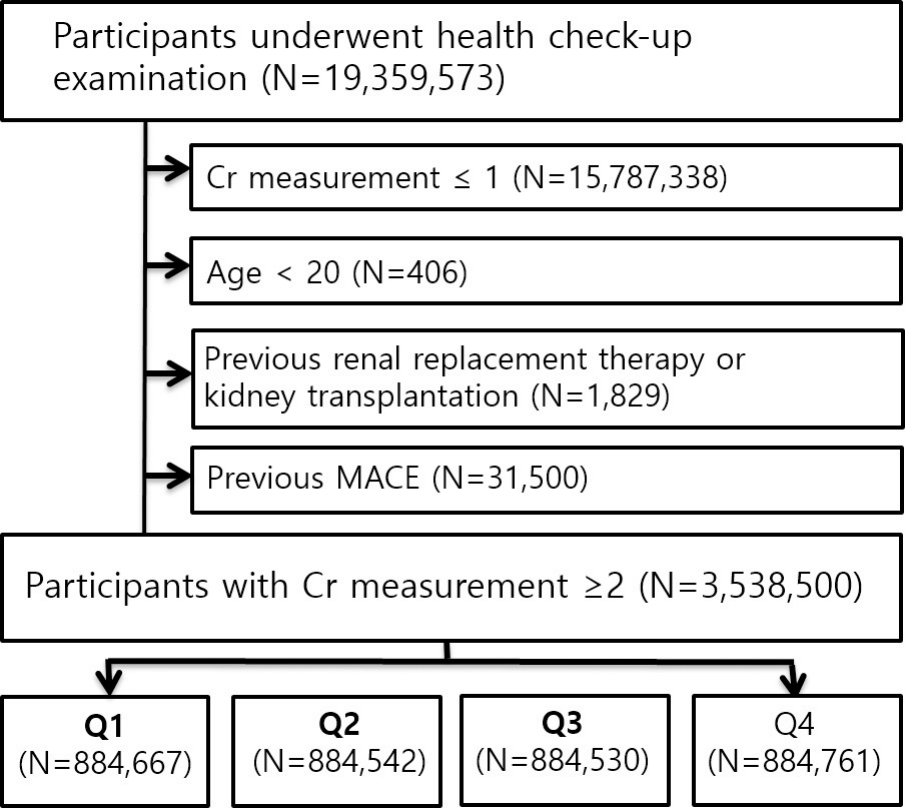
Flow diagram of study participants. The diagram indicates the inclusion and exclusion criteria for the study populations.

All adult participants who underwent the National Health Screening examination with at least three creatinine measurements were screened between 2012 and 2016. Those with 1) missing creatinine values, 2) eGFR under 60 mL/min/1.73 m^2^ at every measurement, 3) a previous diagnosis of cardiovascular disease, or 4) previous kidney transplantation, or renal replacement therapy before of the initial creatinine measurement were excluded (Fig 1). Then, the risks of myocardial infarction (MI), stroke, and death were assessed.

### Data collection

The NHIS dataset contains demographic information (e.g., age, sex, socioeconomic status, underlying diseases), claims data (e.g., prescription history, outpatient clinic visits and hospital admission), and health examination data (e.g., lifestyle questionnaires and laboratory exam results). The comorbidities and diagnosed disease information in the claims data were presented as International Classification of Disease, 10th Revision (ICD-10) codes. Height, weight, and blood pressure were measured by trained clinicians. Laboratory examination results included serum creatinine, the lipid profile, fasting glucose values sampled after overnight fasting, and urinalysis results. Samples were obtained in the NHIS-certified centers, which undergo quality control regularly according to the standard guidelines. Smoking and alcohol consumption status were collected by self-reported questionnaires during the regular health examination.

### Variability in eGFR

The glomerular filtration rate (GFR) was estimated with the Modification of Diet in Renal Disease (MDRD) equation, using the serum creatinine value measured with a non-isotope dilution mass spectrometry (IDMS) assay by the Jaffe kinetic method [16]. To reflect the variation in eGFR values measured more than 3 times through the health examinations, variability independent of the mean (VIM) index was used [17]. It was calculated by the following equation: 100 x standard deviation/mean^β^. β was a regression coefficient, which was a natural logarithm of the standard deviation over the natural logarithm of the mean. The study participants were classified into the four quartiles (Q) of the VIM, and Q4, the highest quartile, represented the participants with the highest eGFR variability.

### Study outcomes

The primary outcome was newly developed MI, stroke, and death. The outcomes were defined with ICD-10 codes (MI; I21 or I22, stroke; I63 or I 64), which were newly issued more than twice in the claims data during the hospital admission or outpatient clinic visits. The death data were obtained from Statistics Korea. All participants were followed up from the initial eGFR measurement to the time of the cardiovascular events, death, or until the end of the follow-up period.

### Statistical analysis

Continuous variables are presented as medians (interquartile ranges), and categorical variables are presented as numbers (percentages). The incidence rate (IR) was calculated by the number of incident cases divided by the entire follow up duration (person-year). Multivariate Cox regression analysis was used to assess the hazard ratios (HRs) and 95% confidence intervals (CIs) of the study outcomes. A multivariate adjusted Cox proportional hazard model was additionally constructed with adjustment variables including age, sex, BMI, smoking, drinking habits, income status, underlying comorbidities and baseline eGFR.

To minimize the potential impact of the annual change in eGFR values on the variability in eGFR, additional analyses were performed after dividing the patients into 3 groups according to the slope of eGFR value: decline, stable and increase group. In addition, subgroup analyses were performed to assess whether mortality increases with elevated eGFR variability according to the underlying diseases and lifestyle habits.

All analyses were performed using SAS version 9.4 (SAS Institute Inc, USA). A P-value under 0.05 was considered statistically significant.

## Results

### Baseline characteristics

The baseline characteristics of the study participants according to the quartiles of eGFR variability are presented in Table 1. Q4 group participants showed the highest VIM index, which represented the highest variability in eGFR. The participants in the Q4 group were older and more frequently diagnosed with hypertension, diabetes, and dyslipidemia (P < 0.001) than the participants in the low variability quartile groups.

**Table 1:** Baseline characteristics of the study participants.

	Q1 (N = 884,667)	Q2 (N = 884,542)	Q3 (N = 884,530)	Q4 (N = 884,761)	P value
Age (years) (N,%)	40.8 ± 10.3	46.2 ± 11.3	43.1 ± 11.2	46.9 ± 11.6	<0.001
Male sex (N,%)	741543 (83.8)	470140 (53.2)	704892 (79.7)	505250 (57.1)	<0.001
Baseline Creatinine	0.9 ± 0.2	0.9 ± 0.2	0.9 ± 0.2	0.9 ± 0.3	<0.001
Baseline eGFR	90.7 ± 15.4	84.2 ± 16.3	89.1 ± 19.0	87.3 ± 24.8	<0.001
eGFR VIM	3.3 ± 2.6	7.2 ± 0.8	11.4 ± 1.4	20.0 ± 7.1	<0.001
Smoking					<0.001
- Never	349566 (39.5)	531927 (60.1)	370654 (41.9)	510224 (57.7)	
- Previous	190354 (21.5)	160587 (18.2)	199291 (22.5)	155663 (17.6)	
- Current	344747 (39.0)	192028 (21.7)	314585 (35.6)	218874 (24.7)	
Drinking (N,%)					<0.001
- Never	277984 (31.4)	401835 (45.4)	304055 (34.4)	413837 (46.8)	
- Current	89985 (10.2)	53737 (6.1)	84288 (9.5)	59714 (6.8)	
Low income	94821 (10.7)	146194 (16.5)	124541 (14.1)	190484 (21.5)	<0.001
Comorbidities (N,%)					
- Hypertension	140649 (15.9)	175072 (19.8)	167712 (19.0)	205221 (23.2)	<0.001
- Diabetes mellitus	52142 (5.9)	64153 (7.25)	64895 (7.3)	77687 (8.8)	<0.001
- Dyslipidemia	135081 (15.27)	165307 (18.69)	153703 (17.4)	194170 (22.0)	<0.001
Body Mass index (Mean ± SD)	24.1 ± 3.3	23.6 ± 3.3	24.1 ± 3.3	24.0 ± 3.3	<0.001
Systolic Blood pressure (Mean ± SD)	122.0 ± 12.8	120.4 ± 13.7	121.8 ± 13.1	121.3 ± 13.7	<0.001
Diastolic Blood pressure (Mean ± SD)	76.8 ± 9.1	75.7 ± 9.5	76.7 ± 9.3	76.1 ± 9.4	<0.001

The data are presented as N (%) or mean ± standard deviation.

Low income was defined as a total income, 20^th^ percentile for the nation.

eGFR = estimated glomerular filtration rate, VIM = variability independent of mean, SD = standard deviation.

### Risk of adverse outcomes according to eGFR variability

**Fig 2:**
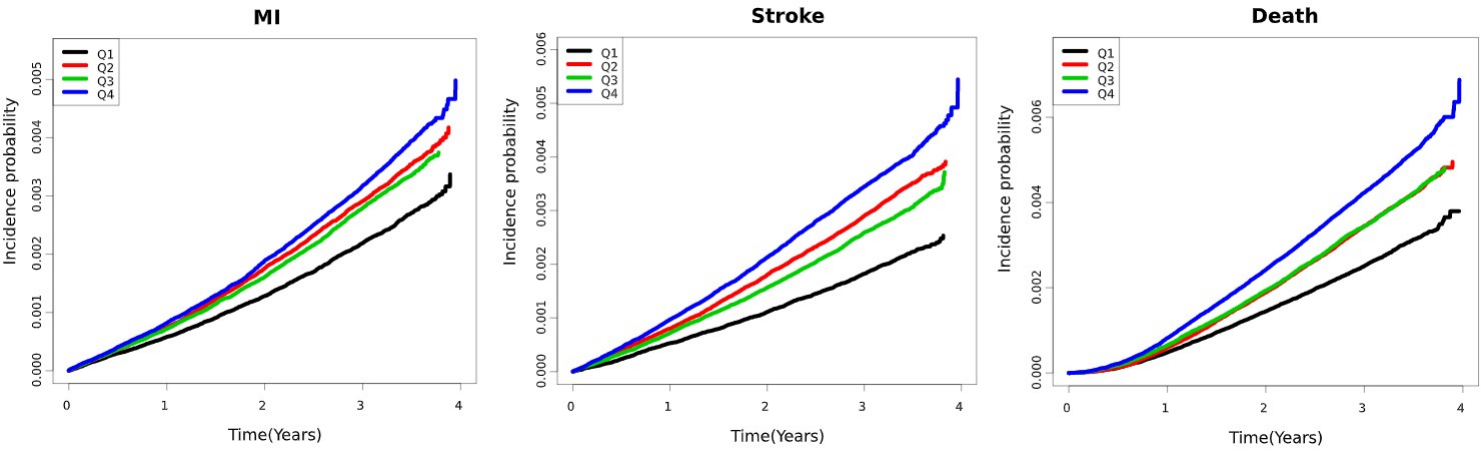
Incidence probability of MI, stroke, and death according to GFR variability. Curves indicate the Kaplan-Meier estimates of the risk of MI, stroke and death by the quartiles of the eGFR variability. The X axis indicates time (years), and the Y axis indicates the incidence probability (%) of the outcomes.

A total of 3,538,500 participants satisfied the inclusion criteria for the outcome analyses. During the median follow up of 3.27 (3.04–3.56) years, 10,697 myocardial infarction (MI) (0.37%), 10,273 stroke (0.30%), and 13,217 death (0.29%) events were observed among all participants. Participants with the highest eGFR variability exhibited overall increased incidence rates and elevated risk of adverse outcomes, compared to those with the lower eGFR variability (Table 2 and Fig 2), namely MI (hazard ratio [HR]: 1.10; 95% confidence interval (CI) 1.04–1.16), stroke (HR: 1.16; 95% CI 1.09–1.23), and death (HR: 1.18; 95% CI 1.12–1.24) The results were consistent after adjusting for potential confounding factors, including age, sex, BMI, drinking, smoking habits, income status, baseline eGFR, and the presence of hypertension, diabetes, and dyslipidemia, except the risk of MI (HR: 1.06; 95% CI 1.00–1.12).

**Table 2:** Association of eGFR quartile and adverse outcomes.

	Follow up duration (Person-year)	Events (N)	Incidence rate (per 1000 person-years)	Model 1 HR (95% CI)	Model 2 HR (95% CI)	Model 3 HR (95% CI)
MI						
Q1	2834001	2137	0.75	1 (reference)	1 (reference)	1 (reference)
Q2	2826814	2810	0.99	1.03 (0.97–1.09)	1.03 (0.98–1.10)	1.04 (0.99–1.11)
Q3	2821839	2671	0.95	1.04 (0.99–1.10)	1.04 (0.99–1.11)	1.04 (0.98–1.10)
Q4	2810893	3079	1.10	1.10 (1.04–1.16)	1.09 (1.03–1.15)	1.06 (1.00–1.12)
P for trend				0.001	0.003	0.114
Stroke						
Q1	2834439	1767	0.62	1 (reference)	1 (reference)	1 (reference)
Q2	2826718	2786	0.99	1.04 (0.98–1.10)	1.05 (0.99–1.11)	1.05 (0.99–1.12)
Q3	2822037	2461	0.87	1.07 (1.00–1.13)	1.06 (1.00–1.13)	1.06 (0.99–1.12)
Q4	2810457	3259	1.16	1.16 (1.09–1.23)	1.15 (1.08–1.22)	1.11 (1.05–1.18)
P for trend				<0.001	<0.001	<0.001
Death						
Q1	2836951	2467	0.67	1 (reference)	1 (reference)	1 (reference)
Q2	2830660	3331	1.18	0.96 (0.91–1.02)	1.00 (0.95–1.05)	1.00 (0.95–1.06)
Q3	2825480	3331	1.18	1.05 (0.99–1.11)	1.05 (1.00–1.11)	1.05 (0.99–1.11)
Q4	2815029	4088	1.45	1.18 (1.12–1.24)	1.17 (1.11–1.23)	1.16 (1.10–1.22)
P for trend				<0.001	<0.001	<0.001

Model 1: Adjusted for age, and sex.

Model 2: Adjusted for age, sex, smoking, baseline eGFR, underlying diabetes.

Model 3: Adjusted for model 2 plus BMI, drinking, income, underlying hypertension, and dyslipidemia.

eGFR = estimated glomerular filtration rate, HR = hazard ratio, CI = confidence interval, MI = myocardial infarction, Q = quartile.

### Risk of adverse outcomes according to creatinine variability

Analyses examining the variability in the serum creatinine values and the risk of adverse outcomes were additionally performed. The variability in the multiple serum creatinine measurement values was also stratified into quartiles using the VIM index. Participants with the highest creatinine variability exhibited an elevated risk of stroke (HR: 1.07; 95% CI 1.01–1.14) and death (HR: 1.22; 95% CI 1.16–1.29), compared to those with lower creatinine variability. (S1 Table).

### The difference in eGFR variability and the annual change in eGFR

To distinguish the independent impact of eGFR variability on the annual change in eGFR, additional analyses were performed after stratifying all study participants according to the eGFR slope into decline, stable, and increase subgroups. The eGFR slope, the percentage of the annual eGFR change, was calculated by the change in absolute eGFR difference over the time interval (total years) between the initial and final eGFR measurements. Within 10%, differences in the slope were grouped in the stable category. Then, the impact of the increased eGFR variability on adverse outcomes was examined, compared to the remaining low eGFR variability. The highest eGFR variability exhibited an independent association with elevated risks of stroke and death, regardless of the annual decline or increase in eGFR (Table 3). However, eGFR variability did not show an independent association with the risk of MI. Increased mortality with high eGFR variability was similarly observed when the slope difference range was changed to 5% (S2 Table).

**Table 3:** Association of the variability quartiles and the outcomes according to the slope of GFR (slope index 10%).

	Variability Quartile	Events (N)	Follow-up duration (person-years)	Incidence rate (per 1000 person years)	Model 1	Model 2	Model 3
MI							
Decline (10%)	Q1-3	2136	2371675	0.90	1 (reference)	1 (reference)	1 (reference)
	Q4	1331	1147050	1.16	1.10 (1.02–1.18)	1.04 (0.96–1.12)	1.02 (0.94–1.09)
Stable	Q1-3	2973	3290320	0.90	1 (reference)	1 (reference)	1 (reference)
	Q4	325	278360	1.17	1.10 (0.98–1.24)	1.09 (0.97–1.22)	1.05 (0.94–1.18)
Increase (10%)	Q1-3	2509	2820661	0.89	1 (reference)	1 (reference)	1 (reference)
	Q4	1423	1385482	1.03	1.02 (0.95–1.09)	1.03 (0.96–1.10)	0.99 (0.92–1.06)
	P for interaction				0.299	0.797	0.653
Stroke							
Decline (10%)	Q1-3	2031	2371730	0.86	1 (reference)	1 (reference)	1 (reference)
	Q4	1510	1146765	1.32	1.20 (1.12–1.28)	1.11 (1.03–1.19)	1.09 (1.01–1.17)
Stable	Q1-3	2790	3290426	0.85	1 (reference)	1 (reference)	1 (reference)
	Q4	309	278335	1.11	0.99 (0.88–1.12)	0.98 (0.87–1.10)	0.95 (0.85–1.07)
Increase (10%)	Q1-3	2193	2821038	0.78	1 (reference)	1 (reference)	1 (reference)
	Q4	1440	1385358	1.04	1.07 (1.00–1.14)	1.06 (0.99–1.14)	1.03 (0.97–1.11)
	P for interaction				0.022	0.048	0.046
Death							
Decline (10%)	Q1-3	2636	2374566	1.11	1 (reference)	1 (reference)	1 (reference)
	Q4	1858	1148839	1.62	1.24 (1.12–1.32)	1.22 (1.14–1.30_	1.20 (1.13–1.28)
Stable	Q1-3	3462	3294387	1.05	1 (reference)	1 (reference)	1 (reference)
	Q4	363	278788	1.30	1.01 (0.91–1.13)	1.03 (0.93–1.15)	1.02 (0.91–1.14)
Increase (10%)	Q1-3	3031	2824138	1.07	1 (reference)	1 (reference)	1 (reference)
	Q4	1867	1387402	1.35	1.13 (1.06–1.19)	1.07 (1.01–1.14)	1.07 (1.01–1.13)
	P for interaction				0.004	<0.001	0.001

Model 1: Adjusted for age, and sex.

Model 2: Adjusted for age, sex, smoking, baseline eGFR, underlying diabetes.

Model 3: Adjusted for model 2 plus BMI, drinking, income, underlying hypertension, and dyslipidemia.

eGFR = estimated glomerular filtration rate, MI = myocardial infarction, Q = quartile.

### *Subgroup* analysis

Moreover, subgroup analyses were performed to assess the association of eGFR variability and all-cause death according to the underlying comorbidities and lifestyle habits. An increased risk of death in the highest quartile of participants was observed in the participants diagnosed with diabetes (HR: 1.24; 95% CI 1.15–1.34) and in the non-smoking participants (HR: 1.19; 95% CI 1.13–1.25) (Table 4) compared to the remaining low variability quartile participants.

**Table 4:** Risk of all-cause death of Q4 participants in subgroups.

Subgroup	aHR (90% CI)	P for interaction
No DM	1.11 (1.06–1.16)	0.046
DM	1.24 (1.15–1.34)	
No HTN	1.10 (1.05–1.16)	0.138
HTN	1.18 (1.12–1.25)	
No Dyslipidemia	1.13 (1.08–1.18)	0.754
Dyslipidemia	1.16 (1.08–1.24)	
Non-Smoking	1.19 (1.13–1.25)	0.012
Smoking	1.06 (0.99–1.12)	
No drinking	1.15 (1.10–1.19)	0.237
Drinking	1.05 (0.93–1.12)	

aHR = adjusted hazard ratio, CI = confidence interval.

Reference: Q1-3 of eGFR variability.

## Discussion

Using a large-scale nationwide population-based dataset, the present study demonstrated that high eGFR variability is associated with an increased risk of MI, stroke, and all-cause death among the general population with preserved renal function. High eGFR variability independently increased the risks of stroke and death, regardless of an accompanying annual eGFR change. A notable association was observed, especially with the eGFR decline. We suggest that the variability index, which reflects the variation in the multiple eGFR measurement values, may be applied as an additional predictor to estimate the risks of cardiovascular complications.

A single measured low eGFR independently predicted cardiovascular disease or mortality in CKD patients [2,18]. In subjects with reduced kidney function, an annual decline or increase in eGFR slope, which reflected the dynamic nature of kidney function over time, better predicted cardiovascular events and death over a single eGFR measurement [8,9,19,20]. However, as eGFR may vary widely even with a slight change in serum creatinine in subjects with preserved kidney function, the clinical significance of eGFR variation needs to be examined. Thus, the present study focused on testing the predictive value of eGFR fluctuation within normal ranges on clinical outcomes among the general population.

Previous reports examined various variability indices and proposed that dynamic variation in metabolic parameters could predict cardiovascular complications in general populations [12]. Variability in cholesterol levels exhibited an association with an increased risk of cardiovascular events and death, indicating that it is mediated by complicated mechanisms related to systemic endothelial injury [14]. High variability in blood pressure was an independent predictor of the increased risk of cardiovascular events or mortality [12,13,21], suggesting that blood pressure variability was associated with atherosclerosis and led to endothelial dysfunction [22,23]. Previous intra-visit blood pressure analysis pointed out that the variation coefficient, which was a frequently used index to express the variability, cannot completely exclude the effect of the average values, and introduced VIM as an alternative index [17]. VIM was calculated by the proportion of the standard deviation over the exponent of the regression coefficient of the mean. The regression coefficient can be obtained from the fitting curves, using the nonlinear regression analysis of the SAS package [17,24]. The application of VIM was expanded to express the variability in numerous metabolic parameters and successfully predicted clinical outcomes [12,14]. Along with the previously assessed indicators, our findings may provide additional evidence that variability in eGFR calculated with the VIM index can be another practical predictor for assessing cardiovascular outcomes.

We proposed that a higher risk of cardiovascular and cerebrovascular events and all-cause death was observed in the subjects with higher variability in eGFR. In this regard, the highest eGFR variability group was older, had lower income, and more frequently had comorbidities than the remaining low quartile groups. Relatively lower socioeconomic status may incur insufficient intake of balanced nutrition and vulnerable medical services, consequently resulting in an increased risk of clinical complications and an alarming mortality rate [25,26]. It can be assumed that individuals susceptible to external factors, including poorly nourished or unbalanced body fluid status, will present unfavorable prognosis. The eGFR variability may be a useful indicator that could reflect the above complex components and contribute to the prediction of adverse clinical outcomes. Further experiments verifying the exact mechanism of eGFR fluctuation on clinical outcomes are necessary to support the present findings.

Regardless of the longitudinal eGFR change, the high variability in eGFR independently increased the risks of stroke and death. The association was more prominent in those with a downward annual change in eGFR. High variability in serum creatinine also increased the risk of stroke and death. The present finding suggests that the general population exhibiting high variation in renal function needs to be carefully monitored, to reduce the risk of stroke and all-cause mortality.

The present study included every eGFR value of the study subjects greater than 60 mL/min/1.73 m^2^ throughout the study period. Hence, it enabled us to test the impact of multiple eGFR measurement valuesin subjects with relatively normal renal function. Therefore, our results may assist clinicians in applying eGFR variability in predicting cardiovascular complications among the general population with preserved kidney function and to distinguish subjects who require early medical attention.

The present study has several strengths. To the best of our knowledge, this is the first study to state the impact of eGFR variability on cardiovascular outcomes and propose that it could be used as a useful parameter to predict complications. Moreover, the study used a large-scale nationwide dataset that contained accurately collected demographic information and laboratory results. However, there are some limitations to be considered when interpreting the results of the study. As it was a retrospective study, it was hard to demonstrate the causality of eGFR variability. Additionally, those who missed the annual or biannual health examination were not included in the study, and potential selection bias might have remained. In addition, as the death data did not contain the information of the main cause of death, it was hard to discriminate the MACE related death from the all-cause death. Further studies are essential to analyze the association of eGFR variability and the risk of MACE related death. Moreover, as the majority of the study participants underwent creatinine measurement when the IDMS-traceable method was not widely validated, the MDRD equation was used to calculate the eGFR value. The bias of the MDRD equation might have led to the increase of false-positive results of eGFR in those with measured GFR>60 mL/min/1.73 m^2^, compared to the eGFR estimated with the CKD-EPI (Chronic Kidney Disease Epidemiology Collaboration) equation [27]. Therefore, participants with falsely low estimated GFR, might left the potential selection bias. Finally, the majority of participants in the NHIS dataset were of Korean ethnicity. Thus, additional studies need to be performed with various ethnicities to demonstrate whether the variability index is generally applicable. Moreover, further prospective trials are warranted to overcome the above limitations.

## Conclusion

In conclusion, high variability in eGFR was associated with an elevated risk of adverse clinical complications. Monitoring eGFR variability may provide additional prognostic benefits among the general population with normal kidney function.

## Supporting information

S1 Table Association of creatinine variability quartile and the adverse outcomes. (DOCX)

S2 Table Association of the variability quartiles and the outcomes according to the slope of eGFR (slope index 5%). (DOCX)
