# Supplementary material for: Impact of variability in estimated glomerular filtration rate on major clinical outcomes: A nationwide population-based study
Source: PLoS One. 2020 Dec 17;15(12):e0244156. doi: 10.1371/journal.pone.0244156 (PMC7746294; doi:10.1371/journal.pone.0244156)
Supplement: S1 Table — (DOCX) [file pone.0244156.s001.docx]

**Supporting information**

**S1 Table.** Association of creatinine variability quartile and the adverse outcomes

|  | Follow up duration (Person-year) | Events (N) | Incidence rate  (per 1000 person years) | Model 1  HR (95% CI) | Model 2  HR (95% CI) | Model 3  HR (95% CI) |
| --- | --- | --- | --- | --- | --- | --- |
| MI |  |  |  |  |  |  |
| Q1 | 2987616 | 2914 | 0.96 | 1 (ref.) | 1 (ref.) | 1 (ref.) |
| Q2 | 2734151 | 2706 | 0.99 | 0.99 (0.94-1.04) | 0.98 (0.93-1.03) | 0.98 (0.93-1.03) |
| Q3 | 2827569 | 2575 | 0.91 | 1.03 (0.98-1.09) | 1.03 (0.97-1.08) | 1.02 (0.97-1.08) |
| Q4 | 2744212 | 2502 | 0.91 | 1.06 (1.00-1.12) | 1.06 (1.00-1.12) | 1.03 (0.97-1.10) |
| P for trend |  |  |  | 0.018 | 0.040 | 0.174 |
| Stroke |  |  |  |  |  |  |
| Q1 | 2987872 | 2628 | 0.88 | 1 (ref.) | 1 (ref.) | 1 (ref.) |
| Q2 | 2734304 | 2547 | 0.93 | 1.01 (0.96-1.07) | 1.01 (0.95-1.06) | 1.01 (0.95-1.06) |
| Q3 | 2827502 | 2558 | 0.90 | 1.08 (1.02-1.14) | 1.08 (1.02-1.14) | 1.07 (1.01-1.13) |
| Q4 | 2743973 | 2540 | 0.93 | 1.09 (1.03-1.16) | 1.10 (1.03-1.16) | 1.07 (1.01-1.14) |
| P for trend |  |  |  | <0.001 | 0.001 | 0.007 |
| Death |  |  |  |  |  |  |
| Q1 | 2991664 | 3202 | 1.07 | 1 (ref.) | 1 (ref.) | 1 (ref.) |
| Q2 | 2737883 | 3354 | 1.23 | 1.08 (1.03-1.14) | 1.06 (1.01-1.12) | 1.06 (1.01-1.11) |
| Q3 | 2831042 | 3315 | 1.17 | 1.23 (1.18-1.30) | 1.18 (1.12-1.24) | 1.17 (1.11-1.23) |
| Q4 | 2747531 | 3346 | 1.22 | 1.33 (1.27-1.40) | 1.24 (1.18-1.31) | 1.22 (1.16-1.29) |
| P for trend |  |  |  | <0.001 | <0.001 | <0.001 |

Model 1: adjusted for age, and sex

Model 2: adjusted for age, sex, smoking, baseline eGFR, underlying diabetes

Model 3: adjusted for model 2 plus BMI, drinking, income, underlying hypertension, and dyslipidemia

MI = myocardial infarction, HR = hazard ratio, CI = confidence interval
