# Supplementary material for: Impact of variability in estimated glomerular filtration rate on major clinical outcomes: A nationwide population-based study
Source: PLoS One. 2020 Dec 17;15(12):e0244156. doi: 10.1371/journal.pone.0244156 (PMC7746294; doi:10.1371/journal.pone.0244156)
Supplement: S2 Table — (DOCX) [file pone.0244156.s002.docx]

**S2 Table.** Association of the variability quartiles and the outcomes according to the slope of eGFR (slope index 5%)

|  | Variability  Quartile | Events (N) | Follow-up duration  (person-years) | Incidence rate  (per 1000 person years) | Model 1 | Model 2 | Model 3 |
| --- | --- | --- | --- | --- | --- | --- | --- |
| MI | | | | | | | |
| Decline (5%) | Q1-3 | 2222 | 2428927 | 0.91 | 1 (reference) | 1 (reference) | 1 (reference) |
|  | Q4 | 1363 | 1158434 | 1.18 | 1.11 (1.03-1.19) | 1.04 (0.97-1.12) | 1.02 (0.95-1.10) |
| Stable | Q1-3 | 2559 | 2948639 | 0.87 | 1 (reference) | 1 (reference) | 1 (reference) |
|  | Q4 | 247 | 226712 | 1.09 | 1.10 (0.96-1.25) | 1.10 (0.96-1.25) | 1.07 (0.93-1.22) |
| Increase  (5%) | Q1-3 | 2837 | 3105089 | 0.91 | 1 (reference) | 1 (reference) | 1 (reference) |
|  | Q4 | 1469 | 1425747 | 1.03 | 1.00 (0.94-1.07) | 1.01 (0.95-1.08) | 0.97 (0.91-1.04) |
|  | P for interaction |  |  |  | 0.144 | 0.547 | 0.401 |
| Stroke | | | | | | | |
| Decline (5%) | Q1-3 | 2132 | 2428972 | 0.88 | 1 (reference) | 1 (reference) | 1 (reference) |
|  | Q4 | 1536 | 1158158 | 1.33 | 1.19 (1.11-1.27) | 1.10 (1.03-1.18) | 1.08 (1.01-1.16) |
| Stable | Q1-3 | 2413 | 2948686 | 0.82 | 1 (reference) | 1 (reference) | 1 (reference) |
|  | Q4 | 240 | 226693 | 1.06 | 0.99 (0.86-1.13) | 0.99 (0.86-1.13) | 0.96 (0.84-1.10) |
| Increase  (5%) | Q1-3 | 2469 | 3105535 | 0.80 | 1 (reference) | 1 (reference) | 1 (reference) |
|  | Q4 | 1483 | 1425606 | 1.04 | 1.05 (0.99-1.13) | 1.05 (0.98-1.12) | 1.02 (0.96-1.09) |
|  | P for interaction |  |  |  | 0.024 | 0.074 | 0.071 |
| Death | | | | | | | |
| Decline (5%) | Q1-3 | 2734 | 2431943 | 1.12 | 1 (reference) | 1 (reference) | 1 (reference) |
|  | Q4 | 1894 | 1160272 | 1.63 | 1.25 (1.17-1.32) | 1.22 (1.15-1.30) | 1.20 (1.13-1.28) |
| Stable | Q1-3 | 3043 | 2952116 | 1.03 | 1 (reference) | 1 (reference) | 1 (reference) |
|  | Q4 | 268 | 227034 | 1.18 | 0.96 (0.85-1.09) | 0.98 (0.87-1.11) | 0.97 (0.86-1.10) |
| Increase  (5%) | Q1-3 | 3352 | 3109032 | 1.08 | 1 (reference) | 1 (reference) | 1 (reference) |
|  | Q4 | 1926 | 1427722 | 1.35 | 1.13 (1.07-1.20) | 1.07 (1.01-1.13) | 1.07 (1.01-1.13) |
|  | P for interaction |  |  |  | 0.001 | <0.001 | <0.001 |

Model 1: adjusted for age, and sex

Model 2: adjusted for age, sex, smoking, baseline eGFR, underlying diabetes

Model 3: adjusted for model 2 plus BMI, drinking, income, underlying hypertension, and dyslipidemia

eGFR = estimated glomerular filtration rate, MI = myocardial infarction, Q = quartile
